# Degradation Mechanism of Mechanical Properties of Concrete in a High Hydraulic Pressure Environment

**DOI:** 10.3390/ma19071430

**Published:** 2026-04-03

**Authors:** Jianmin Du, Xianmin Zhang, Yi Xia, Yongsheng Ji

**Affiliations:** 1 Jiangsu Key Laboratory of Disaster Impact and Intelligent Prevention in Civil Engineering, China University of Mining and Technology, Xuzhou 221116, China; 2 School of Mechanics and Civil Engineering, China University of Mining & Technology, Xuzhou 221116, China; z1216541315@163.com (X.Z.); 19816248045@163.com (Y.X.); jiyongsheng@cumt.edu.cn (Y.J.)

**Keywords:** mechanism degradation, hydraulic pressure, permeation duration, pore water pressure, permeation property

## Abstract

Marine concrete engineering faces severe service environment challenges, including high hydraulic pressure, large stress, and serious penetration. The evaluation of the durability and safety of these structures depends directly on the damage mechanism of concrete materials submitted to high hydraulic pressures. This paper introduced the experimental research on the mechanical properties and the damage mechanism of concrete submitted to high hydraulic pressures. The permeability tests were carried out on concrete specimens under the effect of different hydraulic pressures (1.2 MPa, 2.4 MPa, 3.6 MPa) and durations (10 d, 20 d, 30 d), after which the compressive strength, micro-cracks, and the ultrasonic velocity were obtained and analyzed. The results show that under the effect of sustained high hydraulic pressure, the micro-cracks in concrete increase, the density decreases, and the harmful pores expand, resulting in a degradation in the mechanical properties of concrete. The damage to concrete is more severe at the near end of the hydraulic head than at the far end. The pore water pressure decays gradually with depth inside the concrete and expands inward when the outer layer of concrete is damaged. The conclusions will provide a scientific basis for the safety evaluation of marine concrete engineering.

## 1. General Introduction

With the advancement of deep-earth exploiting strategy, underground concrete engineering is facing severe challenges such as high hydraulic pressure, high stress, and high corrosion, brought by deep strata. The overall safety and durability of underground concrete structures are determined directly by the damage law of the mechanical properties of concrete submitted to high hydraulic pressure. On the one hand, high hydraulic pressure leads to the deterioration of the internal pore structure of concrete, which destroys its anti-permeability and leads to the deterioration of mechanical properties. On the other hand, high hydraulic pressure will accelerate the permeation of harmful ions into the concrete and promote the corrosion of reinforcements. The damage law of material properties of concrete was critically influenced by factors including the mix proportion of concrete, the magnitude of hydraulic pressure, the permeation duration, and so on. The penetration process of chlorides and sulfate ions in concrete is a complex process involving the coupling effect of hydraulic pressure, permeation, and stress. Therefore, the systematic study of the damage evolution mechanism of concrete under high hydraulic pressure in deep underground environments has important theoretical significance and engineering value.

The effect of high hydraulic pressure on the mechanical properties of concrete mainly reflects on the dynamic evolution of porosity and permeability and their complex influence on the mechanical properties of concrete. Porosity and permeability, as critical indicators, directly affect the evolution of the mechanical properties of concrete. Under a high hydraulic pressure environment, the pore water pressure will interact with the external pressure, promote the expansion of gel pores to transition pores, leading to the degradation of concrete strength. Moreover, the non-uniformly saturated state of concrete, where the surface is saturated, and the interior is moist, significantly affects the triaxial strength of concrete due to stress redistribution. The lubrication effect of high hydraulic pressure and chemical penetration further weakens the interfacial bond performance of concrete, reduces the fracture toughness of concrete, and cock aggregate [1,2,3,4]. When the hydraulic pressure is higher, the increase in internal porosity in concrete not only weakens the compressive strength and bulk modulus but also generates a vicious cycle of pore–permeability–erosion due to the intensified connectivity of capillary pores. This promotes the evolution of the pore structure from isolated pores to connected pores, thereby increasing the permeability and leading to strength reduction and interface failure [5,6,7]. There was much research on the additives for concrete to protect against seawater exposure. The additives of nano-particles, such as ZnO and TiO_2,_ into nano-composite coatings can significantly block the penetration of chloride ions and improve the anti-permeability under high hydraulic pressure [8]. Bio-based coatings such as bacterial nanocellulose can effectively enhance the barrier properties of the coating [9]. CaO-based expansion agents can maintain good anti-permeability and mechanical properties while compensating for shrinkage [10]. It is released in the literature that the degradation of concrete strength under high hydraulic pressure environment exhibits an exponential decay, the elastic modulus experiences a two-stage change from a brief enhancement to a long-term sharp drop, and the fracture toughness decreases due to interfacial lubrication and hydraulic splitting [11,12,13]. It is also proven that the damage effect of high hydraulic pressure on concrete exhibits a significant gradient feature. The concrete segment near the water head suffers from greater micro-crack expansion and pore structure deterioration due to direct exposure to high pore water pressure, resulting in a significant decrease in uniaxial compressive strength and elastic modulus, while the damage of the concrete segment far from the water head is obviously weakened [14,15,16].

To quantify the damage of concrete under high hydraulic pressure, ultrasonic detection technology was widely used to analyze the evolution of internal defects through the attenuation of ultrasonic velocity [17,18]. Microscopic characterization techniques indicate that the filling effect of chloride ions and sulfate erosion products in seawater can temporarily block the micro-pores in concrete, slightly increasing the compressive strength in the short term [19,20]. However, the long-term coupled chemical–physical effects will eventually enhance the connectivity of pores in concrete, leading to structural performance deterioration of concrete. High porosity concrete, due to the accelerated ion diffusion and chemical erosion process through connected channels, is more prone to surface spalling and strength degradation [21,22,23,24]. Although existing research methods have made progress in laboratory simulation and microscopic characterization of triaxial tests, most of the research was limited to the discussion of a single environmental factor. Moreover, the existing mechanical models of concrete under high hydraulic pressure environments were proposed based on different hypotheses, such as assuming the dynamic development of cracks as a steady state, which may differ from engineering practice [25,26,27,28].

In order to release the coupling effect of hydraulic pressure and permeation duration on concrete, and clarify the damage mechanism of its mechanical properties, experimental studies were conducted on concrete specimens submitted to various hydraulic pressures (1.2 MPa, 2.4 MPa, 3.6 MPa), and permeation durations (10 days, 20 days, 30 days). The uniaxial compressive strength, peak strain, and elastic modulus of concrete after high hydraulic pressure were recorded and analyzed. The conclusions of this paper will provide a theoretical basis for the safety assessment and durability research of underground concrete engineering.

## 2. Experiment Program

### 2.1. Specimen Preparation

The concrete specimens for the standard permeation tests were truncated cones with a top surface diameter of 175 mm, a bottom surface diameter of 185 mm, and a height of 150 mm. The concrete was mixed with ordinary Portland cement PO.42.5, natural medium sand, and crushed stone with a particle size of 5–20 mm (all these materials are produced in Xuzhou, China). The detailed mix proportion of the concrete is shown in Table 1.

According to the investigation conditions on hydraulic pressures (1.2 MPa, 2.4 MPa, 3.6 MPa) and permeation duration (10 d, 20 d, 30 d), there were in total ten groups of specimens tested. The detailed group number of the specimens is listed in Table 2, in which the group number P1.2-D20 means the specimen submitted to 1.2 MPa hydraulic pressure, and the duration is 20 days.

### 2.2. Permeation Test

**Figure 1:**
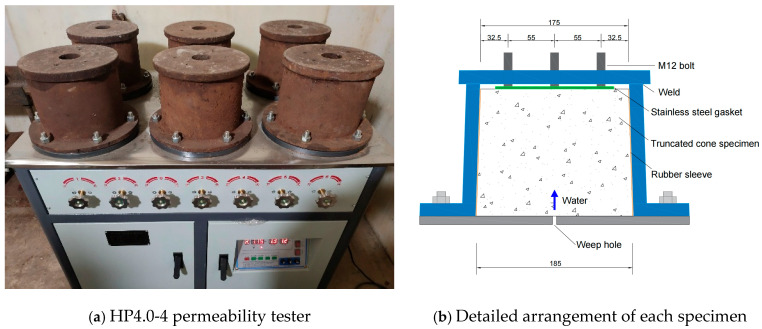
HP4.0-4 permeability tester.

The permeation test was carried out by the designed permeability tester HP4.0-4 (Wuxi Jianyi Instrument and Machinery Co., Ltd., Wuxi, China), shown in Figure 1. The hydraulic pressure applied to the specimens was supplied by the water tank, full of tap water. The steel mold was sealed on top, providing a reacting force to balance the water pressure, so as to avoid large lateral pressures on the specimens. The hydraulic pressure gradient was set separately to 1.2 MPa, 2.4 MPa, and 3.6 MPa.

### 2.3. Mechanical Property Tests of Concrete Before and After Permeation

**Figure 2:**
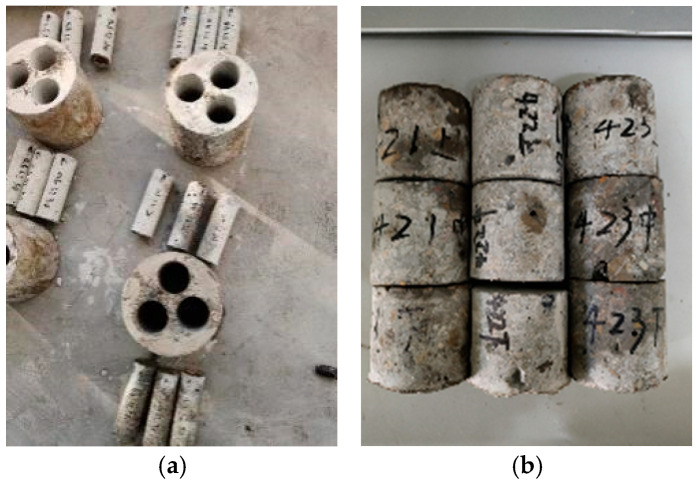
An example of the final blocks from one concrete cone. (**a**) Core samplings from truncated cone; (**b**) three segments of each core sample.

When the objective permeation duration was reached, the specimens were taken out and dried in an oven. Three cylindrical core samples with a diameter of 50 mm and a height of 150 mm were taken from each concrete truncated cone, shown in Figure 2a. And then, the compressive test was carried out for the standard concrete core samples by the YAW-3000 electro-hydraulic servo universal test machine (Jinan Chengyu Testing Equipment Co., Ltd., Jinan, China). By maintaining the loading rate within a reasonable range of 0.03 mm/min, ideal experimental data can be obtained. The average value of strain gauge measurements taken from both ends of the specimen was used as a representative value for the axial compression deformation of the specimen. The pressure measurement is conducted by a JHBU-20T sensor (Jiangsu Donghua Testing Technology Co., Ltd., Taizhou, China), which was connected to a DH3817 data acquisition system (Jiangsu Donghua Testing Technology Co., Ltd., Taizhou, China). The stress–strain curves of concrete specimens were recorded, from which the compressive strength, ultimate strain, and elastic modulus of the concrete submitted to different hydraulic pressures and different durations were discussed.

Then, another three cylindrical core samplings were taken from concrete truncated cones with a diameter of 50 mm and a height of 150 mm, shown in Figure 2a, and then every core sample was divided into three segments (top, middle, and bottom, accounting from the far side to the near side of the hydraulic head), each one 50 mm in height. Thus, nine blocks were taken out of one concrete truncated cone, as shown in Figure 2b.

In the end, the ultrasonic velocity in concrete blocks was measured by the ZBL-U520 non-metallic ultrasonic detector (Beijing ZBL Science and Technology Co., Ltd., Beijing, China) to analyze the density of concrete segments at different distances away from the hydraulic head.

## 3. Mechanical Damage of Concrete Due to High Hydraulic Pressure

### 3.1. Failure Mode of Uniaxial Compression

**Figure 3:**
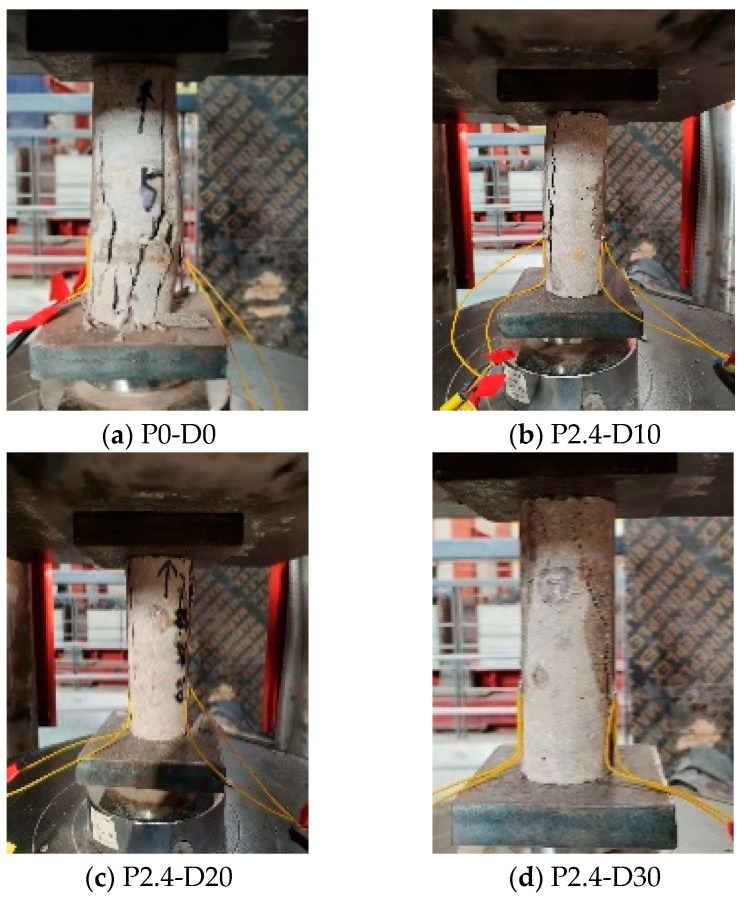
Compressive failure mode of the concrete after different perpetration durations.

The compressive failure mode of the concrete specimens varies with different permeation durations. Take the specimens submitted to 2.4 MPa hydraulic pressure as an example, shown in Figure 3.

The failure mode of all the specimens submitted to hydraulic load or not is similar, with macroscopic cracks through the failure surface, and crushing and spalling occurred on the loading surface.

### 3.2. Stress–Strain Relationship of Concrete Submitted to High Hydraulic Pressure

**Figure 4:**
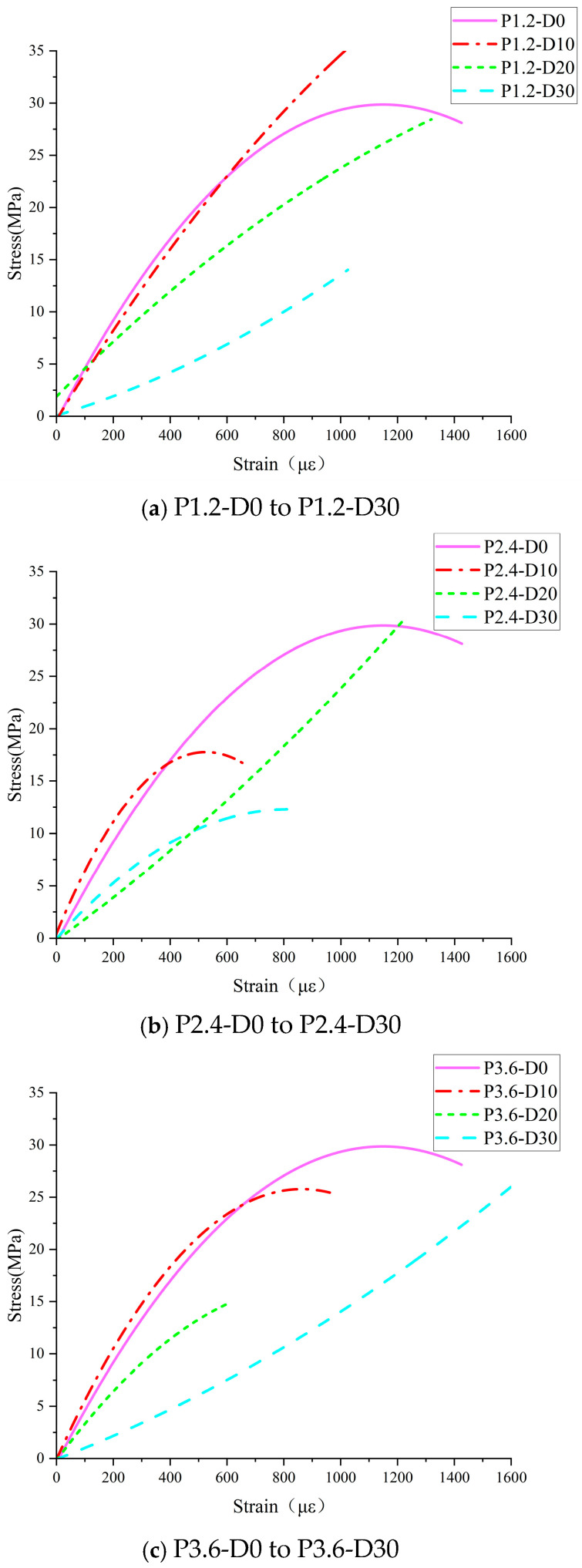
Stress–strain constitutive relationship curve of concrete under various hydraulic pressures.

The stress–strain constitutive relationship curves of the concrete core samplings under various hydraulic pressures were obtained during the compressive tests. In each hydraulic pressure condition, curves of concrete submitted to various durations were compared, as shown in Figure 4.

The results show that the higher the hydraulic pressure, the lower the peak stress of the stress–strain curves, the bigger the ultimate strain, and the lower the slope of the initial elastic stage, which respects the elastic modulus. The specific loading and deformation process can be divided into the following three stages.

During the initial loading stage (σ ≤ 0.4 σ_max_), the stress increased linearly with strain. The internal stress level of the concrete remained relatively low, and the initial cracks were compacted under the compressive load. There was a limited amount of new micro-cracks appearing, and corresponding energy dissipation occurred, but not sufficient to influence the mechanical properties of concrete. The concrete was still in an elastic compressive state.

When the load increased to the stage of 0.4 σ_max_ < σ ≤ 0.8–0.9 σ_max_, the specimen entered the plastic deformation state. The slope of the stress–strain curve gradually decreased, and both the stress and strain of concrete grew, leading to significant development of cracks in the concrete, mainly focused on the aggregate–paste interface. Subsequently, visible cracks emerged on the surface of the specimen. Localized spalling of concrete was observed at the loading surface of the specimen.

During the failure stage (σ > 0.8–0.9 σ_max_), the slope of the stress–strain curve approached zero, and then the descending part appeared. At this stage, the deformation of concrete core samples increased rapidly, with penetrating cracks appearing on their surface and spalling occurring at the ends. In the end, the pressure value dropped rapidly, indicating the failure of the concrete core samplings.

**Figure 5:**
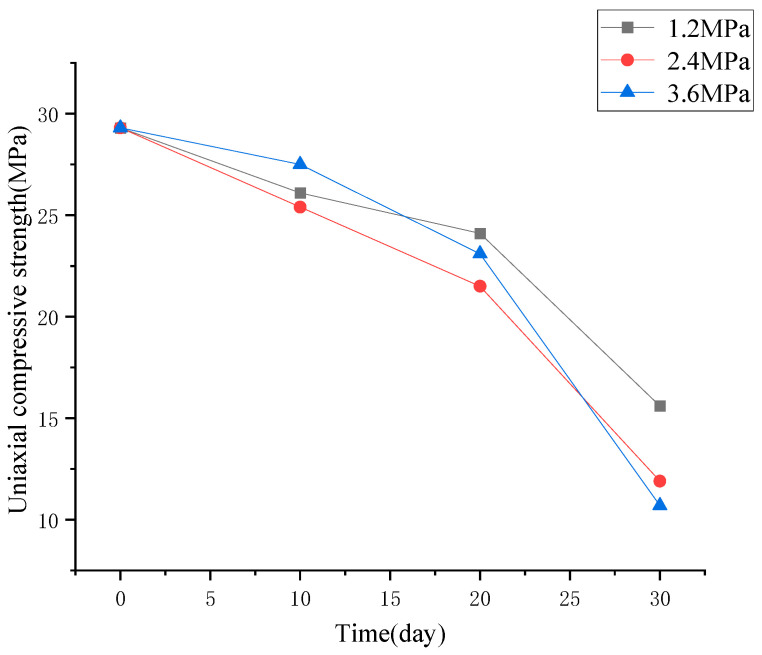
A decrease in compressive strength of concrete after being subjected to various hydraulic pressures.

**Figure 6:**
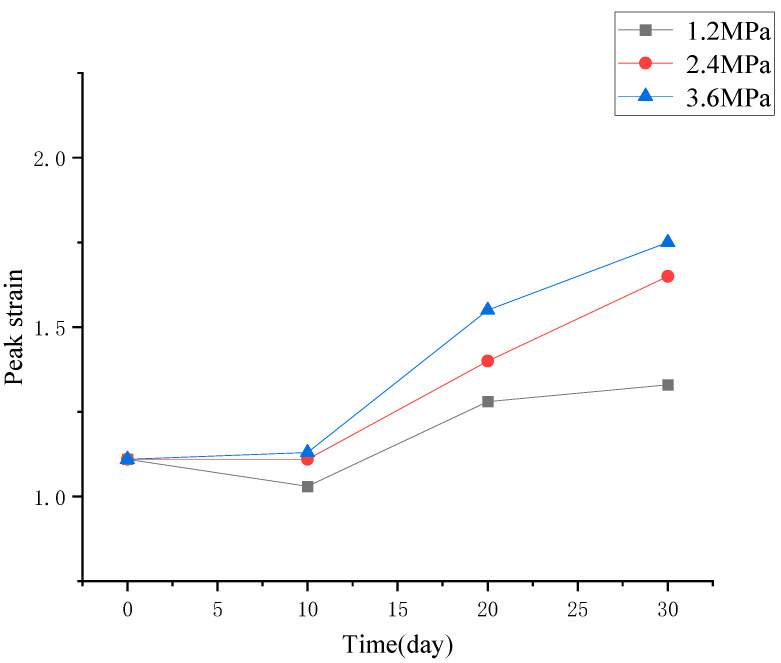
Change in peak strain of concrete after being subjected to different levels of hydraulic pressure.

**Figure 7:**
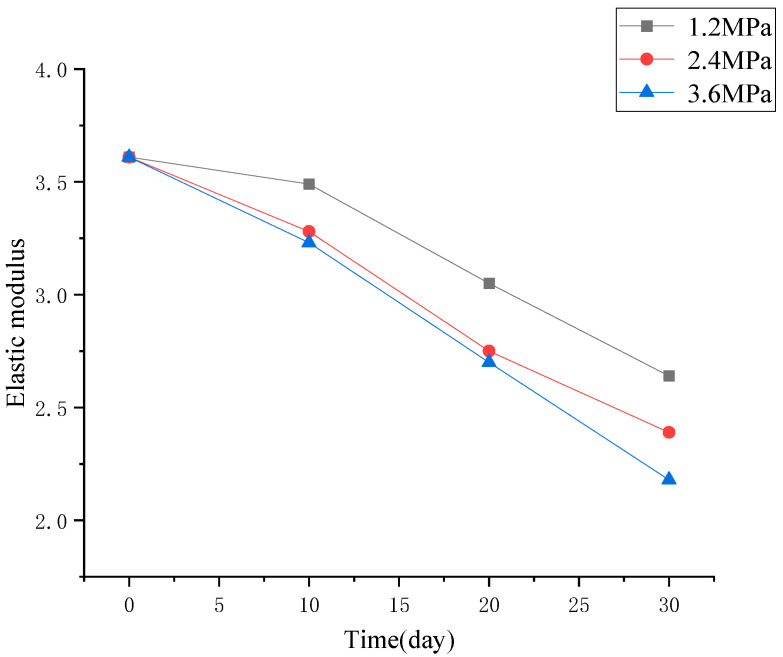
Elastic modulus of concrete after being subjected to different levels of hydraulic pressure.

Based on Figure 4, key parameters for evaluating the deterioration of mechanical properties of concrete, including the change in compressive strength, peak strain, and elastic modulus, were induced and discussed separately, as shown in Figure 5, Figure 6 and Figure 7.

From Figure 5, it can be seen that the compressive strength of concrete submitted to high hydraulic pressure will deteriorate with the increase in hydraulic pressure and the extension of its duration. After 30 days of permeation, the compressive strength of damaged concrete subjected to 1.2 MPa, 2.4 MPa, and 3.6 MPa hydraulic pressure dropped from 29.3 MPa to 15.6 MPa, 11.9 MPa, and 10.7 MPa, respectively, reduced by 46.8%, 59.4%, 63.5%.

From Figure 6 and Figure 7, it can be concluded that the peak strain of concrete submitted to high hydraulic pressure exhibits an increasing trend, while the elastic modulus tends to decline, with the increase in hydraulic pressure and the extension of its duration. Moreover, the higher the hydraulic pressure and the longer the permeation duration, the more obvious the trend was.

### 3.3. Evolution Mechanism of Concrete Pore Structure

**Figure 8:**
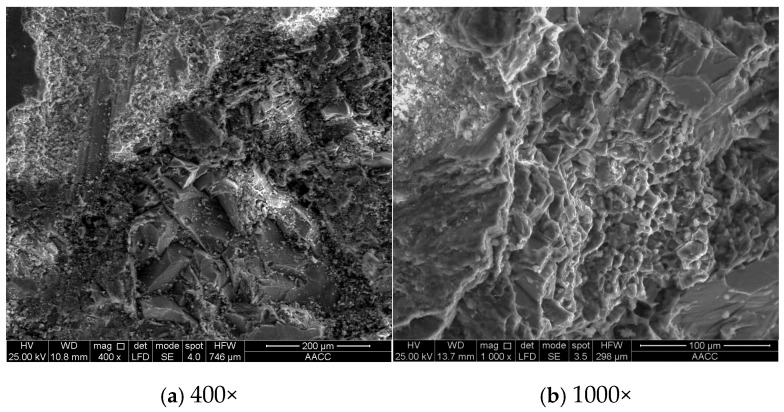
Microstructure of specimen P0-D0.

**Figure 9:**
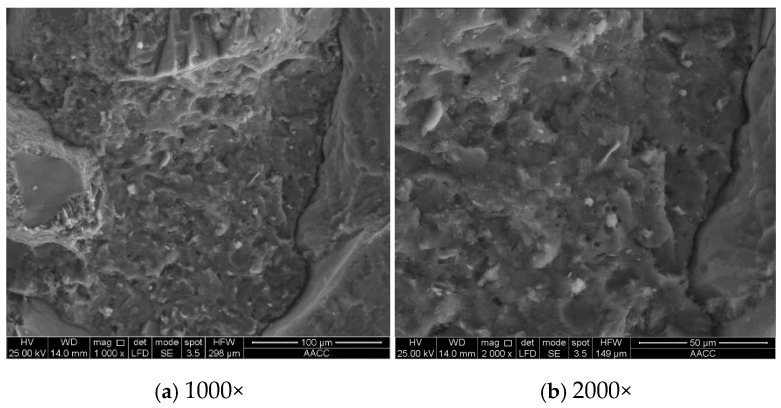
Microstructure of specimen P2.4-D30.

It is concluded by Lim [29] that the concrete specimens subjected to pressure showed increased capillary pores and micro-cracks. To reveal the evolution mechanism of concrete pore structure under high hydraulic pressures, the development of micro-cracks in the specimens under different permeability conditions was observed through a scanning electron microscope (SEM). Combined with the mercury intrusion method, a quantitative characterization of the evolution law of concrete pore structure was proposed. The microstructure of samples taken from the same position of specimen P0-D0 and specimen P2.4-D30 is shown in Figure 8 and Figure 9. The mercury intrusion results, including porosity, proportion of pores, and pore size distribution, are listed in Table 3.

Based on the micro-structural results in Figure 8, the concrete specimen P0-D0 contains only a small number of micro-cracks. In contrast, as observed in Figure 9, specimen P2.4-D30 displayed obvious cracks. A comparison of the microstructure of the two sets of specimens reveals that after being continuously penetrated for 30 days under hydraulic pressure of 2.4 MPa, the internal structure of the concrete underwent noticeable changes, primarily manifested in the formation and expansion of micro-cracks. This phenomenon contributes to the conclusion that high hydraulic pressure environments can accelerate the initiation and propagation of cracks, leading to a significant degradation in the anti-permeability ability and mechanical properties.

It can be seen from Table 3 that the pore structure of concrete before and after permeation is quite different. The porosity of concrete specimens after being submitted to high hydraulic pressure of 2.4 MPa for 30 days increases from 16.7% to 21.5%, and the total pore volume of the pores increases from 0.0767 mL/g to 0.0875 mL/g.

**Figure 10:**
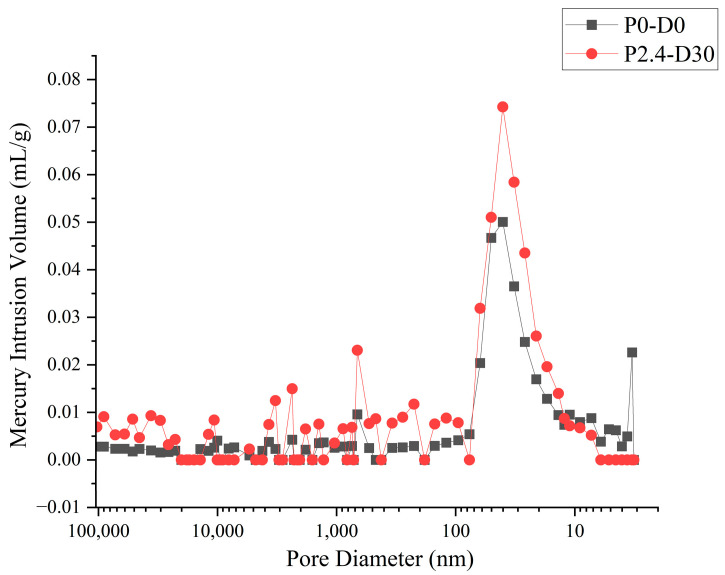
Pore size distribution curve of concrete.

**Figure 11:**
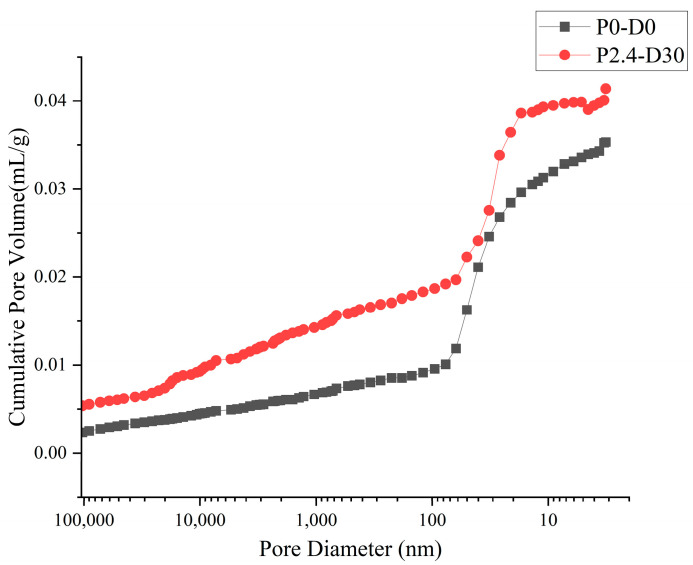
Cumulative pore volume distribution curve of concrete.

Based on the standards and engineering experience, the pores in concrete can be classified into four groups, including harmless pores (diameter < 20 nm), slight-harm pore (20–50 nm), harmful pores (50–200 nm) and hazardous pores (>200 nm). In order to get a clear insight into the change in the pore structure, the pore size distribution diagram and the cumulative pore volume distribution diagram are shown in Figure 10 and Figure 11.

It shows that the specimen submitted to high hydraulic pressure (P2.4-D30) has a larger pore volume for the pores bigger than 20 nm, especially for the harmful pores and hazardous pores, while the harmless pore volume is reduced. The volume ratio of harmful and hazardous pores in specimen P2.4-D30 reached 41.2%, while that of the specimen without pressure is only 24.2%. This proves that high hydraulic pressure makes the harmless small-sized pores expand and connect into bigger pores and change to be hazardous, which damages the inner structure of concrete and decreases its mechanical properties and permeability.

## 4. Influence of Distance Away from Hydraulic Head

The literature shows that the damage zone of concrete moves from the surface to the internal part, as the hydraulic pressure tends to go further into the concrete after the cracking of the surface concrete. This dynamic development process proves that the pore water pressure in concrete is not constant, but grows gradually, making the damage gradually expand from the surface to the interior. The evolution characteristics of concrete mechanical properties over time can be effectively revealed by layered permeability tests.

### 4.1. Influence of Distance from Hydraulic Head on Compressive Strength of Concrete

**Figure 12:**
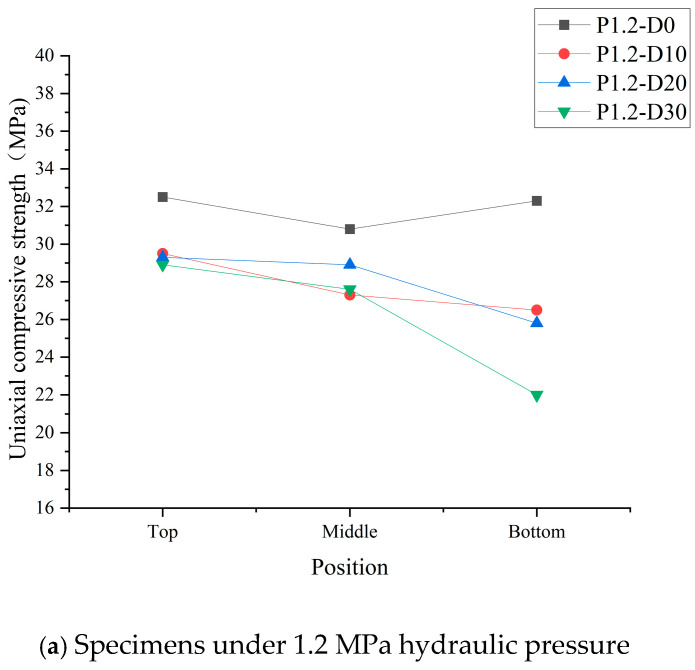

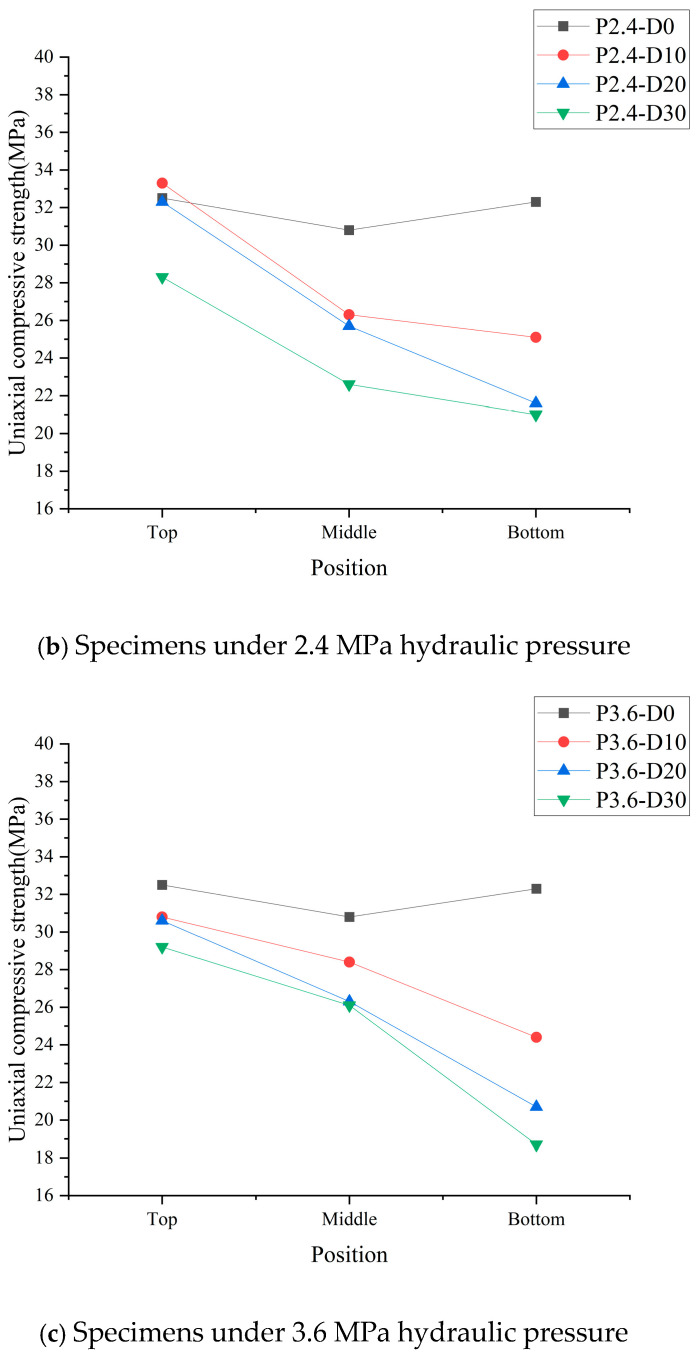
Uniaxial compressive strength of concrete under different hydraulic pressures.

Based on the compression test, the uniaxial compressive strength of concrete segments of the core samples was measured. The compressive strength of the top, middle, and bottom segments, accounting from the far side (top) to the near side (bottom) of the hydraulic head, is shown in Figure 12.

The compressive strength of concrete gradually degrades with the applied hydraulic pressure and duration, besides which it is also influenced by the distance from the hydraulic head. The damage degree of the bottom segment concrete is the most severe, which is the one near the hydraulic head, and that of the top segment, farthest from the hydraulic head, is relatively light. Take specimen P3.6-D30 as an example. The strength of the bottom segment (near the hydraulic head) decreased from 32.3 MPa to 18.7 MPa, with a reduction rate of 44.9%. Whereas that of the top segment (far from the hydraulic head) dropped from 32.5 MPa to 29.2 MPa, resulting in a reduction rate of 10.2%.

### 4.2. The Ultrasonic Velocity of Segment at Different Distances from Hydraulic Head

**Figure 13:**
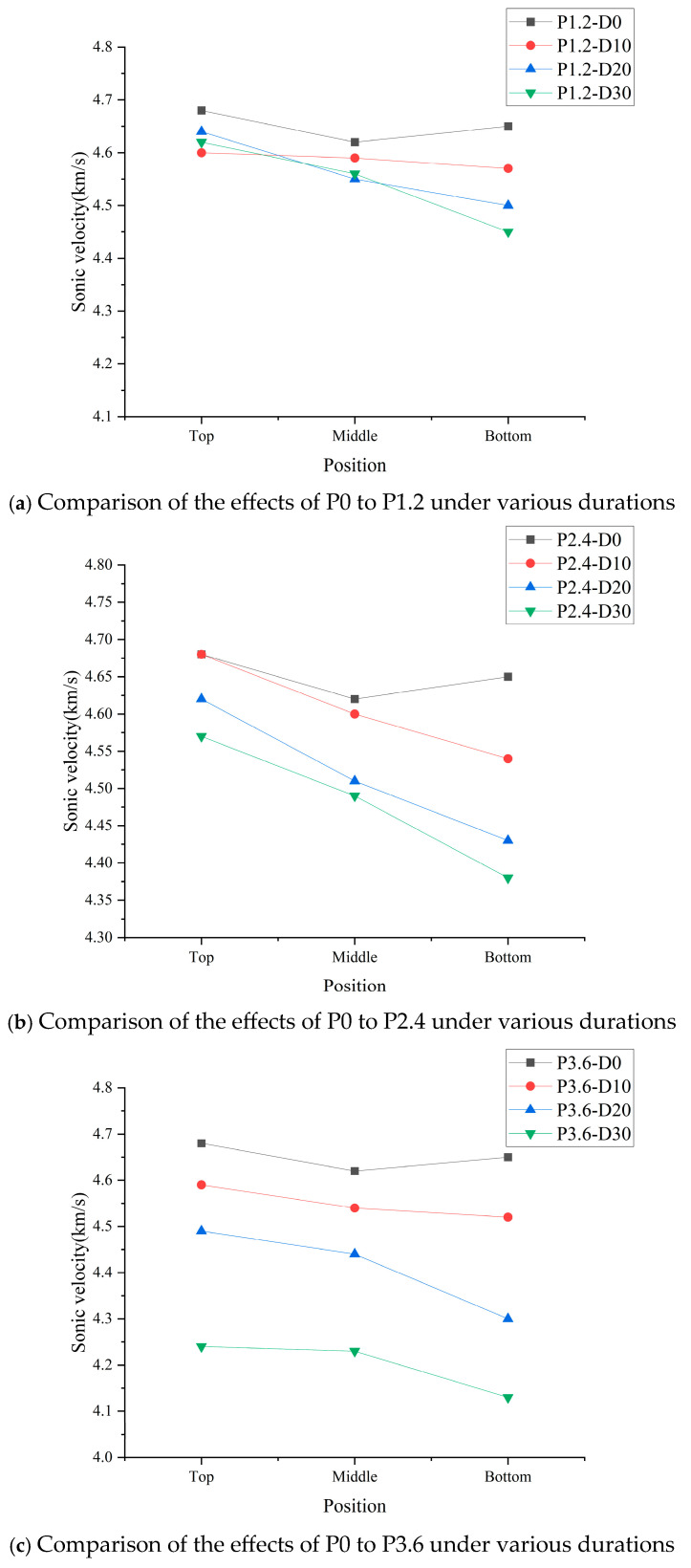
Ultrasonic velocity of concrete after being subjected to various hydraulic pressures.

The propagation time of ultrasonic emission in concrete segments at different distances from the hydraulic head was measured, and the standardized ultrasonic velocity value was obtained, as shown in Figure 13.

It can be seen from Figure 13 that the propagation velocity of the ultrasonic wave exhibits a negative correlation with the permeation duration. This phenomenon happens due to the deterioration of internal pore structures and a progressive reduction in material compactness, caused by the long-term effect of high hydraulic pressure.

The attenuation of the ultrasonic velocity in specimens submitted to higher hydraulic pressure for the same duration is more significant, which can be concluded from the comparison of Figure 13a and Figure 13c. After permeation, the ultrasonic velocity in specimen P1.2-D30 dropped to 4.45 km/s, while that in specimen P3.6-D30, which was submitted to a higher hydraulic pressure, dropped to 4.13 km/s.

Comparing the segments of the same permeation condition, taking P3.6–30 as an example, the ultrasonic velocity in the top segment decreases from 4.68 km/s to 4.24 km/s, and that in the bottom segment decreases from 4.65 km/s to 4.13 km/s, indicating that the damage in concrete near the hydraulic head is more severe than that further from the hydraulic head.

## 5. Conclusions

This study discussed the degradation mechanisms of concrete under high hydraulic pressure and the depth-dependent damage patterns using an experimental method. The following conclusions were obtained.

The uniaxial compressive strength and elastic modulus of ordinary concrete decay with increasing hydraulic pressure and the permeation duration, while peak strain increases. This trend intensifies with strengthened pressure and prolonged exposure.

The high hydraulic pressure moves gradually inwards and causes further destruction of the pore structures of concrete, leading to a decline in the compressive strength and an increase in the ultimate strain of the concrete. The compressive strength of damaged concrete submitted to 1.2 MPa, 2.4 MPa, and 3.6 MPa hydraulic pressure reduced by 46.8%, 59.4%, 63.5% respectively, and the corresponding elastic modulus declined linearly with the extension of duration.

The hydraulic pressure inside the concrete is not constant; it moves gradually to the inside of the concrete, which is similar to the process of sulfate ion permeation. When the concrete specimen is submitted to high hydraulic pressure for a certain duration, the decay of hydraulic pressure with depth inside the concrete is almost linear.

The damage of concrete at the near-end of hydraulic head damage is more pronounced than that at the far-end, as evidenced by the compressive strength of concrete. Moreover, the prolonged permeation duration amplifies the difference in rebound values of the concrete at the two ends of the hydraulic head.

## Figures and Tables

**Table 1:** The mix proportion of concrete.

Water–Cement Ratio	Cement/kg/m^3^	Water /kg/m^3^	Sand /kg/m^3^	Coarse Aggregate /kg/m^3^	Water-Reducing Agent /kg/m^3^
0.50	400	200	536	1084	0.8

**Table 2:** Numbering of the tested specimens.

Specimen Number	Hydraulic Pressure (MPa)	Permeation Duration (Day)
P0-D0	0	0
P1.2-D10	1.2	10
P1.2-D20	1.2	20
P1.2-D30	1.2	30
P2.4-D10	2.4	10
P2.4-D20	2.4	20
P2.4-D30	2.4	30
P3.6-D10	3.6	10
P3.6-D20	3.6	20
P3.6-D30	3.6	30

**Table 3:** Pore structure of concrete.

Specimen Number	Total Pore Volume /mL/g	Total Pore Area /m^2^/g	Middle-Size Pore Volume /nm	Middle-Size Pore Area /nm	Average Pore Size /nm	Porosity /%
P0-D0	0.0767	18.439	35.04	9.43	18.76	16.6531
P2.4-D30	0.0875	23.635	62.65	26.38	49.65	21.4916

## Data Availability

The original contributions presented in this study are included in the article. Further inquiries can be directed to the corresponding author.
